# Multiparametric FDG-PET/MRI of Hepatocellular Carcinoma: Initial Experience

**DOI:** 10.1155/2018/5638283

**Published:** 2018-10-03

**Authors:** Stefanie J. Hectors, Mathilde Wagner, Cecilia Besa, Wei Huang, Bachir Taouli

**Affiliations:** ^1^Translational and Molecular Imaging Institute, Icahn School of Medicine at Mount Sinai, 1 Gustave L Levy Place, New York, NY 10029, USA; ^2^Sorbonne Universités, UPMC, Department of Radiology, Hôpital Pitié-Salpêtrière, Assistance Publique-Hôpitaux de Paris, 47-83 Boulevard de l'Hôpital, 75013 Paris, France; ^3^Department of Radiology, School of Medicine, Pontificia Universidad Católica de Chile, Santiago, Chile; ^4^Advanced Imaging Research Center, Oregon Health & Science University, 3181 SW Sam Jackson Park Rd L452, Portland, OR 97239, USA; ^5^Department of Radiology, Icahn School of Medicine at Mount Sinai, 1 Gustave L Levy Place, New York, NY 10029, USA

## Abstract

**Purpose:**

To compare multiparametric (mp)FDG-PET/MRI metrics between hepatocellular carcinoma (HCC) and liver parenchyma and to assess the correlation between mpMRI and FDG-PET standard uptake values (SUVs) in liver parenchyma and HCC.

**Methods:**

This prospective, institutional review board-approved study enrolled 15 patients (M/F 12/3; mean age 61 y) with HCC. mpMRI including blood-oxygen-level-dependent (BOLD) MRI, intravoxel incoherent motion diffusion-weighted imaging (IVIM-DWI), and dynamic contrast-enhanced-(DCE-) MRI was performed simultaneously with ^18^F-FDG-PET on a 3T PET/MRI hybrid system. Quantitative BOLD, IVIM and DCE-MRI parameters (Tofts model (TM) and shutter-speed model (SSM)), and PET parameters (SUV_mean_ and SUV_max_) were quantified and compared between HCC lesions and liver parenchyma using Wilcoxon signed-rank tests. SUV ratios between HCCs and liver were also calculated (SUV_mean_ T/L and SUV_max_ T/L). Diagnostic performance of (combined) mp-PET/MRI parameters for characterization of HCC was assessed using ROC analysis. Spearman correlations between PET and mpMRI parameters in HCC tumors and liver parenchyma were evaluated.

**Results:**

21 HCC lesions (mean size 4.0 ± 2.4 cm; range 2–13 cm) were analyzed. HCCs exhibited significantly higher arterial fraction (from DCE-MRI) and lower *R*_2_^*∗*^ pre-O_2_ and post-O_2_ (from BOLD-MRI) versus liver parenchyma (*P* < 0.032). The highest diagnostic performance for differentiation between HCC and liver parenchyma was achieved for combined ART SSM and *R*_2_^*∗*^ post-O_2_ (AUC = 0.91). SUV_max_ showed reasonable performance for differentiation of HCC versus liver (AUC = 0.75). In HCC, DCE-MRI parameters *K*^trans^ (TM and SSM) and *v*_e_ TM exhibited significant negative correlations with SUV_max_ T/L (*r* ranges from −0.624 to −0.566; FDR-adjusted *P* < 0.050).

**Conclusions:**

Despite the observed reasonable diagnostic performance of FDG-PET SUV_max_ for HCC detection and several significant correlations between FDG-PET SUV and DCE-MRI parameters, FDG-PET did not provide clear additional value for HCC characterization compared to mpMRI in this pilot study.

## 1. Introduction

Hybrid positron emission tomography/magnetic resonance imaging (PET/MRI) technology is becoming increasingly available [1], with oncologic imaging being one of its major potential applications [2]. MRI offers excellent soft tissue contrast, which serves as an anatomical reference for the PET measurements. In addition, functional MRI techniques can supplement PET-based characterization of tumors. PET scan of tumor glucose metabolism using radioactive fludeoxyglucose (FDG) tracer is a well-established method for the clinical diagnosis and monitoring of various cancers throughout the body [3].

While increasingly being applied simultaneously, relatively little is known about the synergy or potential redundancy between FDG-PET and functional multiparametric MRI (mpMRI) in oncology. Several studies have reported significant correlations between standard uptake values (SUVs) from FDG-PET and mpMRI parameters in different types of cancer [4–7]. In hepatocellular carcinoma (HCC), three independent studies have assessed the correlation between FDG-PET SUVs and functional MRI parameters [8–10]. In two studies that employed separate PET/CT and MRI scans with an interval of at least a couple of days, no significant correlation between FDG-PET SUVs and the apparent diffusion coefficient (ADC) from diffusion-weighted imaging (DWI) was found in HCC [8, 9]. A recent study employing hybrid PET/MRI in 41 patients with liver tumors showed a significant negative correlation between FDG-PET SUV and ADC [10]. Dynamic contrast-enhanced- (DCE-) MRI parameter *K*^trans^ has also shown to be negatively correlated with FDG-PET SUVs in HCC [8].

In addition to DCE-MRI and standard monoexponential DWI, other functional MRI techniques and analysis methods may provide additional information on tumor characteristics. Intravoxel incoherent motion DWI (IVIM-DWI), which allows for simultaneous assessment of tissue diffusion and pseudodiffusion due to capillary blood flow [11], has been recently applied for functional imaging of cancer, including HCC [12–14]. Blood oxygenation level-dependent MRI (BOLD-MRI) provides noninvasive indirect quantitative measurement of the level of hypoxia in tumors by exploiting the paramagnetic properties of deoxyhemoglobin [15]. Even for well-established functional MRI methods, additional tissue properties may be assessed using different approaches of data analysis. While DCE-MRI data are typically modeled using the Tofts model (TM) [16], the shutter-speed model (SSM) includes an additional parameter *τ*_i_, the mean intracellular water molecular lifetime, in the pharmacokinetic modeling to account for the kinetics of cross cell membrane water exchange in the extravascular space. This parameter has been suggested to be associated with tissue metabolic activity [17].

The association of mpMRI metrics with histopathological and gene expression markers of HCC has been recently studied by our group [18]. We would now like to assess the correlation of mpMRI parameters with measurements of tumor metabolism quantified with FDG-PET. The combination of mpMRI with FDG-PET using a PET/MRI system yields comprehensive measurements of tumor molecular, morphological, and functional properties, potentially leading to a better understanding of tumor characteristics. Knowledge of the relationship between functional MRI parameters and FDG-PET tumor metabolism measurements may potentially further improve imaging-based characterization of tumors and aid in tumor diagnosis, staging, and treatment stratification.

The goals of this preliminary study were (1) to compare mpMRI metrics and FDG-PET SUVs between HCC and liver parenchyma in HCC patients undergoing simultaneous PET/MRI and (2) to assess the relationships between mpMRI and FDG-PET SUV parameter values in HCC lesions and liver parenchyma.

## 2. Materials and Methods

### 2.1. Patients

This single-center prospective study was compliant with the Health Insurance Portability and Accountability Act and approved by the Institutional Review Board of the Icahn School of Medicine at Mount Sinai. Written informed consent was obtained from all subjects. From January 2014 to August 2016, 15 consecutive patients (M/F 12/3, mean age 61 years (range 49–77 years)) with HCC were enrolled. HCC was diagnosed based on routine imaging by two radiologists in consensus (observer 1 (CB), a radiologist with 6 years of experience in abdominal MRI and 1 year of experience in nuclear medicine, and observer 2 (MW), a radiologist with 5 years of experience in abdominal MRI), according to the Organ Procurement and Transplantation Network (OPTN) criteria [19]. All patients had chronic liver disease with various etiologies (chronic hepatitis C (*n* = 7), chronic hepatitis B (*n* = 5), nonalcoholic steatohepatitis (*n* = 1), alcoholic steatohepatitis (*n* = 1), and cryptogenic cirrhosis (*n* = 1)). Three patients underwent previous HCC treatment using transarterial chemoembolization or yttrium-90 radioembolization (range 110–276 days before the PET/MRI examination). Three patients had pathological evaluation of the HCC lesion(s) within three months before or after the PET/MRI exam (one biopsy 28 days before PET/MRI, and two resections 8 and 15 days after the PET/MRI).

### 2.2. PET/MRI Acquisition

The PET/MRI acquisition was performed using a 3.0T hybrid system (Biograph mMR, Siemens Healthineers, Erlangen, Germany). The system is equipped with a 32-channel spine and flexible body array coil for MRI signal reception and 56 lutetium oxyorthosilicate-avalanche photodiode (LSO-APD) PET detector blocks. Subjects were asked to fast for 6 hours prior to the examination to eliminate effects of postprandial glucose levels on portal blood flow [20]. Approximately one hour before the PET/MRI examination, an intravenous dose of 5.18 MBq/kg ^18^F-FDG was administered to the subjects. PET data were acquired during the MRI acquisition and were corrected for attenuation using a Dixon-based method. PET images were reconstructed to 127 axial images with a field-of-view of 72 cm, matrix size 172 × 172, and slice thickness 2.03 mm. In addition to IVIM-DWI, BOLD, and DCE-MRI, the MRI acquisition consisted of axial and coronal T_2_-weighted turbo-spin echo (HASTE) imaging, axial dual-echo chemical shift imaging, 3D T_1_-weighted imaging before and at a delayed phase (approximately 4 minutes) after injection of a gadolinium contrast agent, and postcontrast axial fat-suppressed T_2_-weighted imaging.

The MRI parameters for the IVIM, BOLD, and DCE-MRI protocols are listed in Table 1. For the BOLD examination, the *R*_2_^*∗*^ acquisitions were performed during a single breath-hold before and at the end of a respiratory oxygen challenge of 10–15 minutes. The oxygen (100% O_2_) was delivered through a Hudson nose and mouth mask (Westmed, Responsive Respiratory, St. Louis, MO). BOLD and IVIM were both acquired before contrast injection, with the IVIM images acquired during the oxygen challenge. The DCE-MRI acquisition was performed during free breathing and consisted of 80 frames of dynamic 3D fast low-angle shot (FLASH) acquisitions at a temporal resolution of 4 s. A half dose (0.05 mmol/kg) of gadobenate dimeglumine (Multihance, Bracco Diagnostics Inc.) followed by a 25 ml saline flush was administered intravenously at a rate of 3 ml/s 8 seconds after the start of the acquisition. Half dose of the contrast agent was used to reduce saturation effects in the DCE-MRI acquisition.

### 2.3. PET Analysis

Observer 1 performed the analysis of the PET images on a dedicated MIMvista workstation (version 6.6; MIM Software Inc., Cleveland, OH). All tumor and nontumoral liver regions were defined by careful correlation with the diagnostic MRI scans. FDG uptake was determined by assessment of the maximal and mean SUV (SUV_max_ and SUV_mean_) in 2D single-slice regions of interest (ROIs) in the liver parenchyma and HCC lesions. For the HCC lesions, ROIs were drawn as large as possible, to encircle the highest tracer activity of each tumor, with guidance from MRI images for anatomical reference. For normal liver regions, two circular ROIs of approximately 2 cm^2^ each were drawn, one in the right lobe and one in the left lobe, and at a location where no tumor was detected on other images. The SUV_max_ of normal liver was defined as the highest SUV_max_ of the two ROIs drawn on normal liver. The SUV_mean_ of normal liver was defined as the mean value of the SUV_mean_ of the two ROIs. An HCC lesion was considered FDG-avid if the SUV_mean_ value was higher than that in the liver parenchyma.

### 2.4. MRI Analysis

Observer 2 performed the ROI analysis of the MRI images, with reference of the ROIs drawn on the PET images. Lesion size was recorded by measuring the largest diameter of the tumor in the axial plane on the delayed postcontrast T_1_-weighted images. Single-slice ROIs were placed in the liver parenchyma and HCC lesions on the DCE-MRI, IVIM, and BOLD images. The ROIs were matched as closely as possible with the PET ROIs.

### 2.5. DCE-MRI Analysis

Prior to pharmacokinetic modeling, motion correction was performed on the DCE-MRI images using a 3D rigid registration algorithm in an open-source image analysis software package (FireVoxel, CAI2R, New York University, New York, NY, USA). The ROI placement for the DCE-MRI images was done in the same FireVoxel software. For determination of the vascular input function, ROIs were drawn in the portal vein on the registered images and in the abdominal aorta at the level of the celiac trunk on the unregistered images [21]. In addition, single-slice ROIs were drawn in the liver parenchyma and the HCC lesion(s) of each patient. The dynamic signal intensity (SI) curves averaged for each ROI were exported, and further analysis was done using custom-written scripts in MATLAB (version R2016b, MathWorks, Natick, MA, USA). The quantitative analysis was performed by observer 3 (SH), an MRI physicist with 3 years of experience. The SI curves were converted to dynamic longitudinal relaxation rate *R*_1_ curves with the spoiled gradient recalled echo (SPGR) equation using precontrast *R*_1_ values from a separate Look–Locker acquisition within the same acquisition protocol. The TM and SSM were subsequently fitted to the dynamic *R*_1_ curves using a nonlinear least-squares fitting algorithm. As vascular input for the modeling, a combination of the aortic and portal venous curves was used according to the following formula: *R*_1,I_= ART*∗R*_1,AIF_ + (1- ART)*∗R*_1,VIF_(*t*- *τ*_VIF_), in which ART is the arterial fraction, *R*_1,I_ is the vascular input *R*_1_ curve, *R*_1,AIF_ is the *R*_1_ curve in the aorta ROI, *R*_1,VIF_ is the *R*_1_ curve in the portal vein ROI, and *τ*_VIF_ is a delay between the arterial and venous input curves [22]. For both TM and SSM modeling, *v*_e_ was constrained to a value between 0 and 1 and *K*^trans^ to a value between 0 and 3 min^−1^. Other parameters needed as input for the modeling included the contrast agent's relaxivity at 3.0T (6.3 mM^−1^·s^−1^ [23]) and the blood hematocrit value for which a fixed value of 0.45 was used. The modeling yielded parameter estimates for transfer constant *K*^trans^ (TM and SSM), extravascular extracellular fraction *v*_e_ (TM and SSM), wash-out rate constant *k*_ep_ (TM and SSM), *τ*_i_ (SSM), and ART (TM and SSM).

### 2.6. IVIM DWI Analysis

For the IVIM DWI analysis, ROIs were drawn in the HCC lesions and liver parenchyma on the diffusion-weighted images using OsiriX (version 5.8, Pixmeo, Bernex, Switzerland) software. The biexponential IVIM model [11] was fit to the mean signal curve in the ROI at different *b* values to estimate the pseudodiffusion coefficient *D*^*∗*^, diffusion coefficient D, and the perfusion fraction PF. The fitting procedure was performed in MATLAB using a Bayesian algorithm [24]. In addition, the apparent diffusion coefficient (ADC) was determined by calculation of the slope of a linear fit through the logarithmic signal data at the different *b*-values. For ADC estimation, only *b*-values of 0 and >150 s/mm^2^ were included to avoid a disproportionate effect of perfusion-influenced measurements on the ADC [14].

### 2.7. BOLD MRI Analysis

Similar to the IVIM analysis, ROIs of the BOLD MR images were drawn in OsiriX. Using a custom-written script in MATLAB, a monoexponential model was fit to the mean signal intensity curves at the different echo times in the liver and HCC ROIs to estimate *R*_2_^*∗*^. This analysis was done for the acquisitions before and after O_2_ challenge, and Δ*R*_2_^*∗*^ was determined (*R*_2_^*∗*^ post-O_2_-*R*_2_^*∗*^pre-O_2_).

### 2.8. Statistical Analysis

Statistical analysis was performed in MATLAB. Nonparametric tests were used, given the small sample size. Wilcoxon signed-rank tests were used to evaluate the differences in PET/MRI parameters between the liver parenchyma and HCC lesions. In patients with more than one HCC lesion, the average of the parameters from multiple HCC lesions was taken for statistical analysis. Receiver operating characteristic (ROC) analysis was performed to assess the diagnostic performance of each of the mp-PET/MRI parameters for differentiation between liver and HCC tissues. Logistic regression with stepwise forward selection of features using Wald tests was performed to determine the optimal combination of features for separation of HCC from liver parenchyma. Spearman correlation analysis was done to assess the correlation between the PET and MRI parameters. This analysis was done separately for the liver parenchyma, HCC lesions, and treatment-naive HCC lesions. In addition, the Spearman correlation analysis with MRI parameters was done for SUV ratios between HCC lesions and liver parenchyma (SUV_mean_ T/L and SUV_max_ T/L). Spearman correlation was also employed for assessment of correlation between PET/MRI parameters and lesion size derived from MRI. *P* values of the correlation analyses were corrected for multiple tests using a false discovery rate (FDR) correction. For all tests, a *P* value lower than 0.05 was considered statistically significant.

## 3. Results

### 3.1. Lesions

21 HCC lesions (average size 4.0 cm; range 2–13 cm) were identified in 15 patients. The distribution of number of lesions per patient was as follows: 1 lesion (*n* = 10), 2 lesions (*n* = 4), and 3 lesions (*n* = 1). All lesions were included for the DCE-MRI analysis. Three lesions were excluded from the IVIM analysis because of severe artifacts (*n* = 2) or because the lesion was not in the field-of-view (FOV) of the IVIM acquisition (*n* = 1), resulting in 18 lesions in 14 patients for the IVIM analysis. Two lesions were excluded from the BOLD analysis, because they were located outside the FOV of the acquisition, resulting in 19 lesions in 15 patients for the BOLD analysis. Of the included lesions, 5 lesions were previously treated and exhibited various degrees of necrosis (<10% (*n* = 2), 30% (*n* = 1), 70% (*n* = 1), and 90% (*n* = 1)), which were determined through interpretations of the MR images. Of the 21 HCC lesions, 11 (52%) were FDG-avid.

### 3.2. PET/MRI Quantification

**Figure 1:**
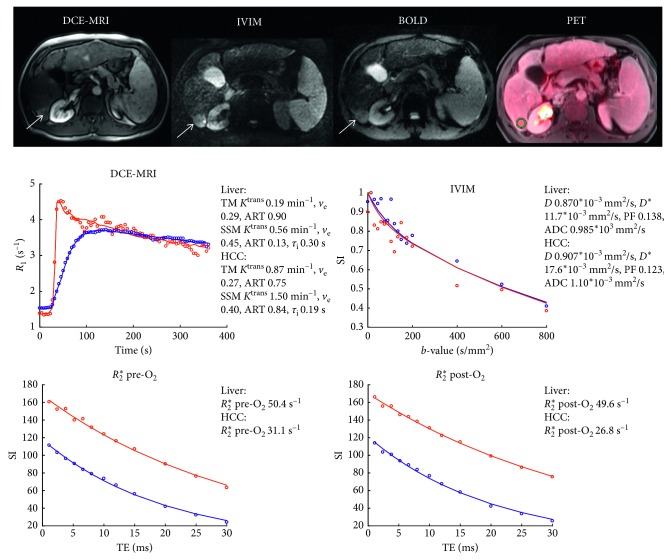
A 56-year-old male patient with cirrhosis secondary to chronic HCV and HCC. DCE-MRI, IVIM (*b* = 400), and BOLD (TE = 30 ms, pre-O_2_) images and PET overlay on anatomical T_2_-weighted image demonstrate 2.7 cm HCC in the right liver lobe (white arrows). Plots of the DCE-MRI, IVIM, and BOLD data points (open circles) and fits (solid lines; SSM fit shown for DCE-MRI) are displayed in the panels at the bottom of the figure for liver (blue) and HCC (red) ROIs. The fitted parameters are shown next to the plots. The HCC lesion showed nonavid FDG uptake (SUV_mean_ 1.57 and SUV_max_ 1.99) and high perfusion/permeability as measured by DCE-MRI.

**Figure 2:**
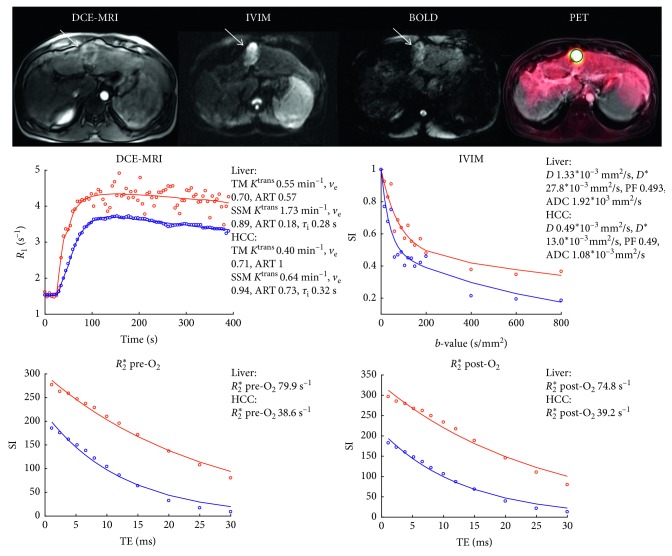
A 51-year-old male patient with cirrhosis secondary to chronic HBV and HCC. DCE-MRI, IVIM (*b* = 400), and BOLD (TE = 30 ms, pre-O_2_) images and PET overlay on anatomical T_2_-weighted image demonstrate 3.8 cm HCC in the left liver lobe (white arrows). Plots of the DCE-MRI, IVIM, and BOLD data points (open circles) and fits (solid lines; SSM fit shown for DCE-MRI) are displayed in the panels at the bottom of the figure for liver (blue) and HCC (red) ROIs. The fitted parameters are shown next to the plots. The HCC lesion showed avid FDG uptake (SUV_mean_ 6.06 and SUV_max_ 7.80) and relatively low perfusion/permeability as measured by DCE-MRI.

Representative PET/MRI images and DCE-MRI, IVIM, and BOLD curves for patients with nonFDG-avid and FDG-avid HCC lesions are shown in Figures 1 and 2, respectively. ROI parameter values in the liver parenchyma and HCC lesions and results of the ROC analysis are displayed in Table 2. For the DCE-MRI parameters, significantly higher ART values were observed in the HCC lesions compared to the liver parenchyma for both the TM and SSM analysis. In addition, a significantly lower *R*_2_^*∗*^ was observed in the HCC lesions compared to liver parenchyma, both before and after the oxygen challenge. The IVIM and PET SUV parameters did not show significant differences between liver and HCC, except for a trend toward higher SUV_max_ in HCC (FDR-adjusted P = 0.091). For SUV_max_, a reasonable AUC of 0.75 was found for differentiation of liver versus HCC, with a sensitivity and specificity of 53.3% and 100%, respectively. The highest diagnostic performance for differentiation between liver and HCC for individual parameters was found for ART SSM (AUC = 0.81). Logistic regression identified the combination of ART SSM and *R*_2_^*∗*^ post-O_2_ as optimal for differentiation between liver and HCC. For this combination, an AUC of 0.91 was found for detection of HCC versus liver parenchyma.

### 3.3. Correlation between FDG-PET and mpMRI

**Figure 3:**
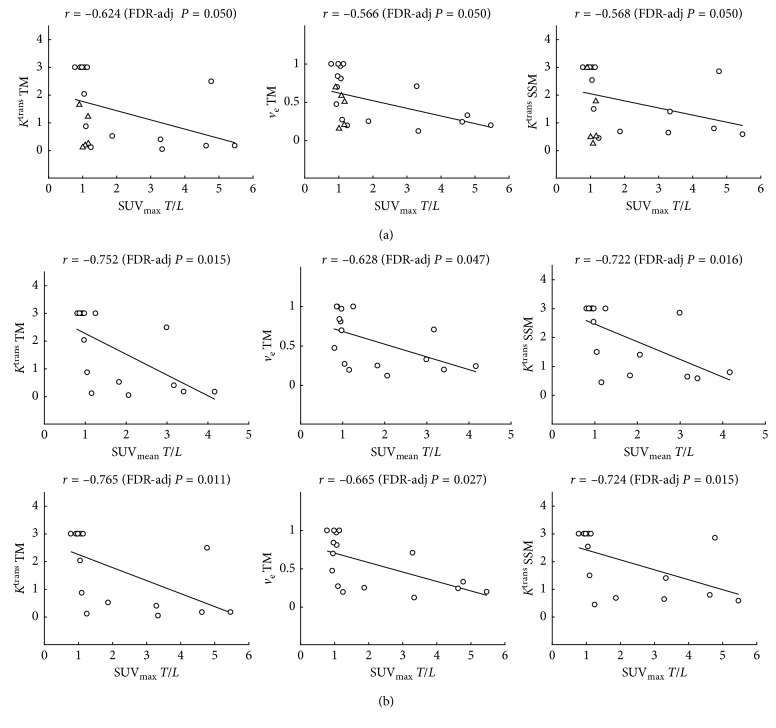
(a) Correlation plots between SUV_max_ T/L (i.e., ratio between SUV_max_ values in HCC vs. liver) and DCE-MRI parameters transfer constant from Tofts model (*K*^trans^ TM), extravascular extracellular fraction from Tofts model (*v*_e_ TM), and transfer constant from shutter-speed model (*K*^trans^ SSM). Treatment-naive HCC lesions are shown as circles and treated HCC lesions are shown as triangles. (b) Correlation plots of SUV_mean_ T/L (top) and SUV_max_ T/L with DCE-MRI parameters *K*^trans^ TM, *v*_e_ TM, and *K*^trans^ SSM in treatment-naive HCC lesions only. The correlation coefficient and corresponding FDR-adjusted *P* values are shown in the top right corner of each plot.

No significant correlations between FDG-PET and mpMRI parameters were observed in the liver parenchyma. In HCC lesions, mpMRI parameters also did not correlate with unnormalized SUVs (*P* > 0.514). However, several significant correlations were observed between DCE-MRI parameters and FDG-PET SUVs in HCC when normalizing the SUVs in HCC to those in liver parenchyma (Figure 3). Specifically, significant negative correlations were observed between *K*^trans^ (TM and SSM) and SUV_max_*T*/*L* (*r* range −0.624 to −0.568, FDR-adjusted *P*=0.050) and between *v*_e_ TM and SUV_max_*T*/*L* (*r* = −0.566, FDR-adjusted *P*=0.050). IVIM-DWI and BOLD parameters did not show significant correlations with FDG-PET parameters (FDR-adjusted *P* > 0.235). When only treatment-naive lesions were analyzed, SUV_mean_ T/L exhibited additional significant correlations with *K*^trans^ (TM and SSM) and *v*_e_ TM (*r* range −0.752 to −0.628, FDR-adj *P* < 0.047; Figure 3(b)). None of the mp FDG-PET/MRI parameters correlated with lesion size (*P* > 0.463).

## 4. Discussion

While PET/MRI is increasingly being used for the characterization of cancer, its applications in clinical oncology may grow even further if the synergy and divergence between functional MRI and PET can be demonstrated for various cancer types. In this study, we quantified functional mpMRI and FDG-PET parameters in HCC and liver parenchyma and assessed correlations between the two techniques. Knowledge of the relationship between functional mp-PET/MRI parameters in HCC may potentially improve HCC characterization and treatment stratification. We found reasonable diagnostic performance of SUV_max_ for differentiation of HCC versus liver, although better characterization was observed when using combined mpMRI parameters. In addition, several significant correlations between FDG-PET SUVs and DCE-MRI parameters were observed in HCC.

The quantitative MRI and PET parameter values in liver and HCC obtained in this study are consistent with those reported in previous studies [9, 14, 22]. The significantly higher ART in HCC versus liver agrees with the known phenomenon that perfusion of HCC lesions is dominated by arterial flow, while the liver is mainly supplied by the portal vein [25]. The lower *R*_2_^*∗*^ in HCC versus liver is in accordance with a previous study [15]. As expected, only 52% of HCC lesions showed avid FDG uptake. HCC lesions generally show weak FDG uptake, potentially due to the high dephosphorylating enzyme activity in hepatocytes and well-differentiated HCC cells, leading to excretion of FDG from the cells [26]. In addition, glucose transporter activity is known to be weak in HCC [26].

Improved diagnostic performance for differentiation of HCC versus liver was seen when using a combination of mpMRI parameters ART SSM and *R*_2_^*∗*^ post-O_2_. FDG-PET SUVs did not show additional value for detection of HCC versus liver. Other nonFDG PET markers, such as ^11^C acetate and ^18^F choline, potentially show increased avidity in HCC lesions [26, 27].

Several significant correlations were found between PET and DCE-MRI parameters in HCC lesions, when normalizing the HCC SUVs to SUVs in the liver parenchyma. The rationale for normalization to reference tissue is that SUV measurements are calculated using body weight, assuming an equal distribution of the radioactive tracer throughout the entire body. However, FDG does not accumulate in the fatty tissues in fasting state, making conventional SUV measurements sensitive to body fat percentage [28]. Normalization to reference tissue potentially eliminates this confounding factor and was recently employed in a study for correlation between DWI and FDG-PET/CT in HCC [9]. The negative correlation between *K*^trans^ and FDG-PET SUV_max_ in HCC lesions has been observed previously in a study where separate MRI and FDG-PET/CT examinations were performed in HCC patients [8]. While this correlation is counterintuitive, as one may expect that high-grade, highly cellular tumors are well perfused and highly metabolic, it may be explained by the fact that tumor progression can be faster than the development of new vasculature, leading to hypoxic conditions [29]. The main metabolic pathway of highly proliferative tissues, including tumors, is glycolysis, in both aerobic and anaerobic conditions [30]. This is in contrast to other tissues, which metabolize using the more energy-efficient pathway of oxidative phosphorylation in aerobic conditions [30]. The pathway and degree of metabolism in tumors is thus not directly dependent on the amount of perfusion and hypoxia. The negative correlation between *K*^trans^ and SUV in HCC is therefore likely not a causal relationship, but a direct observation that *K*^trans^ decreases and SUV increases, respectively, in high-grade HCC lesions [8]. The significant correlations of FDG-PET SUVs with *v*_e_ in HCC are probably also related to tumor progression with lower extravascular extracellular space in highly cellular tumors. Additional significant correlations between DCE-MRI and FDG-PET SUVs were seen when only treatment-naive HCC lesions were included in the analysis, which indicates that treatment-induced biological effects may influence the association between glucose metabolism and perfusion.

FDG-PET SUVs were not significantly correlated with IVIM-DWI and BOLD parameters. Two studies have also shown a lack of correlation between ADC and FDG-PET SUV in HCC [8, 9], while a more recent study in HCC by Kong et al. showed the opposite [10]. These conflicting results suggest that diffusion is not directly correlated to glucose metabolism. In addition to cellularity, the ADC value is also sensitive to other biological properties, including necrosis. The absence of correlations between FDG-PET SUVs and BOLD in HCC suggest that hypoxia and tumor metabolism are not directly associated. This may be explained by the fact that tumors generally exhibit high metabolism, regardless of oxygenation status [30]. Nevertheless, care must be taken in the interpretation of the BOLD measurements. The BOLD acquisitions are known to be influenced by blood volume, flow, and vessel geometry [31]. The chaotic vascular structure in tumors complicates the interpretation of BOLD MRI data in tumors.

Though it has been suggested that the SSM-unique *τ*_i_ parameter is a marker of cellular metabolic activity (17–19), no significant correlations were observed in this study between *τ*_i_ and FDG SUVs. This could be related to the fact that FDG-PET measures cellular uptake of glucose, whereas *τ*_i_ is mainly dictated by Na^+^, K^+^-ATPase activity sustained by ATP production (18), a downstream effect of glucose uptake. In addition, the relatively short TR in the DCE-MRI measurements may also have reduced the sensitivity of the DCE-MRI acquisition to water exchange kinetics [32, 33], and affected the precision of *τ*_i_ quantification. Future studies with improved DCE-MRI sensitivity to the effect of water exchange are needed to investigate the potential utility of SSM DCE-MRI for assessment of tumor metabolism.

The DCE-MRI analysis could be further optimized by improving the AIF determination. In our study, we observed that several DCE-MRI fits converged to *K*^trans^ = 3 min^−1^, which was the upper limit set for the fitting algorithm. Upon observation of these fits, we found that the AIF peak was relatively low in those cases. Apparent reduction of the AIF peak is a known phenomenon and may occur due to susceptibility artifacts from high-contrast agent concentrations or due to low temporal resolution of the DCE-MRI acquisition [34]. While we intentionally administered half dose of contrast agent to reduce saturation effects, susceptibility effects may still have occurred particularly at the relatively high-field MRI system (3.0T) used in our study. In addition, in some cases, the temporal resolution of 4 seconds may not have been fast enough to capture the bolus peak. Several AIF correction techniques have been described, which may reduce the effect of saturation on the AIF quantification [34, 35].

Overall, while several correlations were observed between DCE-MRI parameters and FDG SUVs in HCC, the absence of correlations in the liver and the finding that the majority of the assessed mpMRI, including all BOLD and IVIM-DWI parameters, did not significantly correlate with FDG values in HCC suggest that mpMRI and FDG-PET provide complementary information on liver (tumor) tissue status. However, the exact role of FDG-PET for liver and HCC characterization remains to be investigated.

Our study has several limitations. First, the sample size was small in this preliminary study. Second, no comparison between PET/MRI and pathology could be performed, as pathological confirmation is unnecessary in typical cases, and was available only in 3 patients. Third, not all lesions were treatment-naive. Fourth, the slice thickness was different for the different MRI techniques and the reconstructed PET images, leading to differences in the amount of tumor tissue included in the ROIs. Last, we performed single-slice analysis of the images, because the BOLD acquisition did not cover the entire tumor in several large tumors.

In conclusion, despite the observed reasonable diagnostic performance of FDG-PET SUV_max_ for HCC detection and several significant correlations between FDG-PET SUV and DCE-MRI parameters, FDG-PET did not provide clear additional value for HCC characterization compared to mpMRI in this pilot study. The utility of hybrid FDG-PET/MRI in HCC should be assessed in a larger study.

## Figures and Tables

**Table 1:** MRI acquisition parameters.

	IVIM	BOLD	DCE-MRI
Sequence type	2D SS-EPI	2D MGRE	3D FLASH
Acquisition plane	Axial	Axial	Axial
TE (ms)	75	1.1, 2.4, 3.8, 5.2, 6.6, 8.0, 10.0, 12.0, 15.0, 20.0, 25.0, 30.0	1
TR (ms)	One respiration^*∗*^	249	2.9
FA (°)	90	18	11
*b* values (s/mm^2^)	0, 15, 30, 45, 60, 75, 90, 105, 120, 135, 150, 175, 200, 400, 600, 800	—	—
Number of averages	1, 1, 1, 1, 1, 1, 1, 1, 1, 1, 1, 1, 1, 2, 3, 4	1	1
FOV (mm^2^)	360 × 270	360 × 270	360 × 270
Matrix	128 × 96	512 × 384	384 × 288
Slice thickness (mm)	7	7	4.5
Number of slices	20	5	44
Acceleration factor	2	2	4
Acquisition time (min:s)	08:00	0:15	0:04 per dynamic

BOLD,  blood oxygenation level-dependent; DCE-MRI,  dynamic contrast-enhanced MRI; EPI,  echo planar imaging; FA,  flip angle; FLASH,  fast low-angle shot; FOV,  field-of-view; IVIM,  intravoxel incoherent motion; MGRE,  multigradient recalled echo; TE,  echo time; TR,  repetition time. ^*∗*^IVIM acquisition was respiratory triggered using a navigator echo.

**Table 2:** Average parameter values (mean ± SD) and diagnostic performance of multiparametric FDG-PET/MRI parameter values for differentiation between liver parenchyma and HCC lesions.

	Parameter	Liver	HCC^*∗*^	FDR-adj *P*	AUC	Threshold	Sens (%)	Spec (%)
DCE-MRI	*K* ^trans^ TM (min^−1^)	1.07 ± 0.94	1.62 ± 1.27	0.285	0.61	2.33	46.7	86.7
	*v*_e_ TM	0.46 ± 0.29	0.58 ± 0.31	0.466	0.60	0.51	60.0	73.3
	*k*_ep_ TM (min^−1^)	2.96 ± 2.98	2.66 ± 1.89	0.924	0.51	2.05	73.3	60.0
	ART TM	0.50 ± 0.36	0.85 ± 0.19	**0.032**	0.78	0.60	86.7	66.7
	*K* ^trans^ SSM (min^−1^)	1.61 ± 0.90	1.91 ± 1.14	0.617	0.55	2.54	53.3	80.0
	*v*_e_ SSM	0.58 ± 0.30	0.69 ± 0.29	0.448	0.59	0.91	40.0	80.0
	*k*_ep_ SSM (min^−1^)	3.80 ± 3.16	3.10 ± 1.80	0.629	0.52	5.05	40.0	93.3
	ART SSM	0.33 ± 0.33	0.74 ± 0.29	**0.006**	0.81	0.53	86.7	80.0
	*τ*_i_ (s)	0.27 ± 0.25	0.24 ± 0.17	0.978	0.51	0.32	33.3	80.0

IVIM	*D* (10^−3^ mm^2^/s)	1.34 ± 0.62	1.10 ± 0.27	0.586	0.60	1.51	35.7	92.8
	*D* ^*∗*^ (10^−3^ mm^2^/s)	27.8 ± 23.1	33.6 ± 26.5	0.586	0.62	32.7	50.0	78.6
	PF	0.30 ± 0.16	0.31 ± 0.14	0.870	0.53	0.17	92.9	21.4
	ADC (10^−3^ mm^2^/s)	2.02 ± 1.49	1.59 ± 0.46	0.587	0.57	1.93	50.0	85.7

BOLD	*R*_2_^*∗*^ pre-O_2_ (s^−1^)	85.6 ± 53.4	50.8 ± 18.0	**0.016**	0.74	79.9	46.7	93.3
	*R*_2_^*∗*^ post-O_2_ (s^−1^)	87.8 ± 51.6	50.2 ± 20.4	**0.016**	0.79	46.0	93.3	66.7
	Δ*R*_2_^*∗*^ (s^−1^)	2.47 ± 5.98	−1.14 ± 9.07	0.420	0.58	3.70	40.0	80.0

FDG-PET	SUV_mean_	2.01 ± 0.34	2.88 ± 1.31	0.448	0.70	2.56	46.7	100
	SUV_max_	2.40 ± 0.52	3.93 ± 2.02	0.091	0.75	3.35	53.3	100

Multiparametric	ART SSM + *R*_2_^*∗*^ post-O_2_	0.13 ± 0.08	0.38 ± 0.17	**<0.001**	0.91	0.27	73.3	100

The *P* values originate from Wilcoxon signed-rank tests. Significant *P* values (*P* < 0.05) are shown in bold. The number of lesions analyzed per method was as follows: DCE-MRI, 21 HCC lesions in 15 patients; IVIM, 18 HCC lesions in 14 patients; BOLD, 19 lesions in 15 patients. ^*∗*^Represents the average of parameter values from multiple HCC lesions in patients with more than one lesion. ADC,  apparent diffusion coefficient; ART,  arterial fraction; AUC,  area under the curve; *D*,  diffusion coefficient; *D*^*∗*^,  pseudodiffusion coefficient; FDR,  false discovery rate; *k*_ep_,  rate constant; *K*^trans^,  transfer constant; PF, perfusion fraction; *R*_2_^*∗*^,  transverse relaxation rate; SSM,  shutter-speed model; SUV,  standard uptake value; *τ*_i_, mean intracellular water molecule lifetime; TM,  Tofts model and *v*_e_,  extravascular extracellular volume fraction.

## Data Availability

The data used to support the findings of this study are available from the corresponding author upon request.
